# Supplementary material for: In situ architecture of Opa1-dependent mitochondrial cristae remodeling
Source: EMBO J. 2024 Jan 15;43(3):391–413. doi: 10.1038/s44318-024-00027-2 (PMC10897290; doi:10.1038/s44318-024-00027-2)
Supplement: Supplementary file 1 — Table EV1 [file 44318_2024_27_MOESM1_ESM.pdf]

Table EV1 | Summary of data acquisition and image processing for cryo-ET data in this study.

Sample		WT	Opa1-OE	l-Opa1*	s-Opa1*	Opa1-KO
**Cryo-FIB milling**	**Microscope**	Aquilos Cryo-FIB, FEI – Thermo Fisher Scientific	Aquilos Cryo-FIB, FEI – Thermo Fisher Scientific	Aquilos Cryo-FIB, FEI – Thermo Fisher Scientific	Aquilos Cryo-FIB, FEI – Thermo Fisher Scientific	Aquilos Cryo-FIB, FEI – Thermo Fisher Scientific
**Acquisition settings**	**Microscope**	Titan Krios Gi3 FEI, Thermo Fisher Scientific	Titan Krios Gi3 FEI, Thermo Fisher Scientific	Titan Krios Gi3 FEI, Thermo Fisher Scientific	Titan Krios Gi3 FEI, Thermo Fisher Scientific	Titan Krios Gi3 FEI, Thermo Fisher Scientific
	**Voltage (KeV)**	300	300	300	300	300
	**Detector**	Gatan K3 IS	Gatan K3 IS	Gatan K3 IS	Gatan K3 IS	Gatan K3 IS
	**Energy filter**	Gatan BioQuantum K3	Gatan BioQuantum K3	Gatan BioQuantum K3	Gatan BioQuantum K3	Gatan BioQuantum K3
	**Slit width (eV)**	20	20	20	20	20
	**Super-resolution mode**	Yes	Yes	Yes	Yes	Yes
	**Å/pixel**	2.757	2.565	2.757	2.757	2.757
	**Defocus (μm)**	-3.5 to -5.0	-3.5 to -5.0	-3.5 to -5.0	-3.5 to -5.0	-3.5 to -5.0
	**Acquisition scheme**	-70/70, 2°, Dose-symmetric	-70/70, 2°, Dose-symmetric	-70/70, 2°, Dose-symmetric	-70/70, 2°, Dose-symmetric	-70/70, 2°, Dose-symmetric
	**Total dose**	~90 - 120	~90 - 180	~90 - 180	~90 - 120	~90 - 120
	**Dose rate (e-/Å/sec)**	~ 2.5 – 3.5	~ 2.5 – 3.5	~ 2.5 – 3.5	~ 2.5 – 3.5	~ 2.5 – 3.5
	**Frame number**	6	6	6	6	6
	**Number of tomograms**	33	7	27	22	11
**Image processing**	**Frame alignment and dose weighting**	*framealign*, IMOD	*framealign*, IMOD	*framealign*, IMOD	*framealign,* IMOD	*framealign,* IMOD
	**Tilt series alignment**	IMOD	IMOD	IMOD	IMOD	IMOD
	**WBP**	IMOD	IMOD	IMOD	IMOD	IMOD
	**Denoising**	Topaz	Topaz	Topaz	Topaz	Topaz
	**3D-segmentation**	Amira	Amira	Amira	Amira	Amira
	**3D-rendering**	Amira	Amira	Amira	Amira	Amira
