# Supplementary material for: In situ architecture of Opa1-dependent mitochondrial cristae remodeling
Source: EMBO J. 2024 Jan 15;43(3):391–413. doi: 10.1038/s44318-024-00027-2 (PMC10897290; doi:10.1038/s44318-024-00027-2)
Supplement: Supplementary file 2 — Appendix [file 44318_2024_27_MOESM2_ESM.pdf]

Appendix

In situ architecture of Opa1-dependent mitochondrial cristae remodeling

Michelle Y. Fry, Paula P. Navarro, Pusparanee Hakim, Virly Y. Ananda, Xingping Qin, Juan C. Landoni, Sneha Rath, Zintis Inde, Camila Makhlouta Lugo, Bridget E. Luce, Yifan Ge, Julie L. McDonald, Ilzat Ali, Leillani L. Ha, Benjamin P. Kleinstiver, David C. Chan, Kristopher A. Sarosiek and Luke H. Chao

Table of Content – Appendix

Appendix Figures

Appendix Figure S1 **Page 2**
Appendix Figure S2 **Page 3-4**
Appendix Figure S3 **Page 5**
Appendix Figure S4 **Page 6**
Appendix Figure S5 **Page 7**
Appendix Figure S6 **Page 8-9**

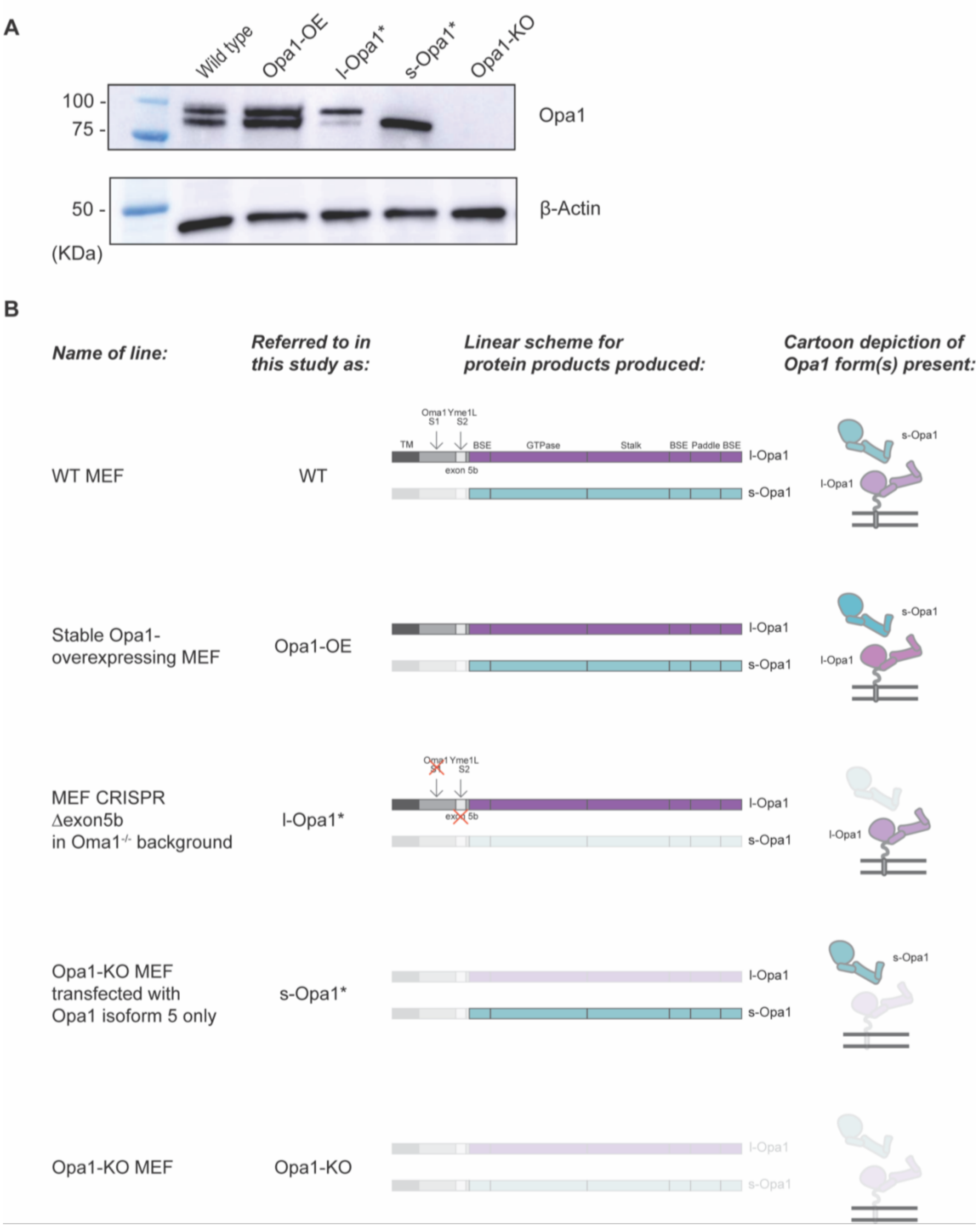

Appendix Figure S1 | Presence of Opa1 forms per MEF cell line.

A. (Top) Western blot detection of Opa1 forms in indicated MEF cell lines using Opa1 antibody. (Bottom) Actin was used as loading control.

B. Genetic schematic and cartoon depictions of Opa1 forms present in MEF cell lines used in this study.

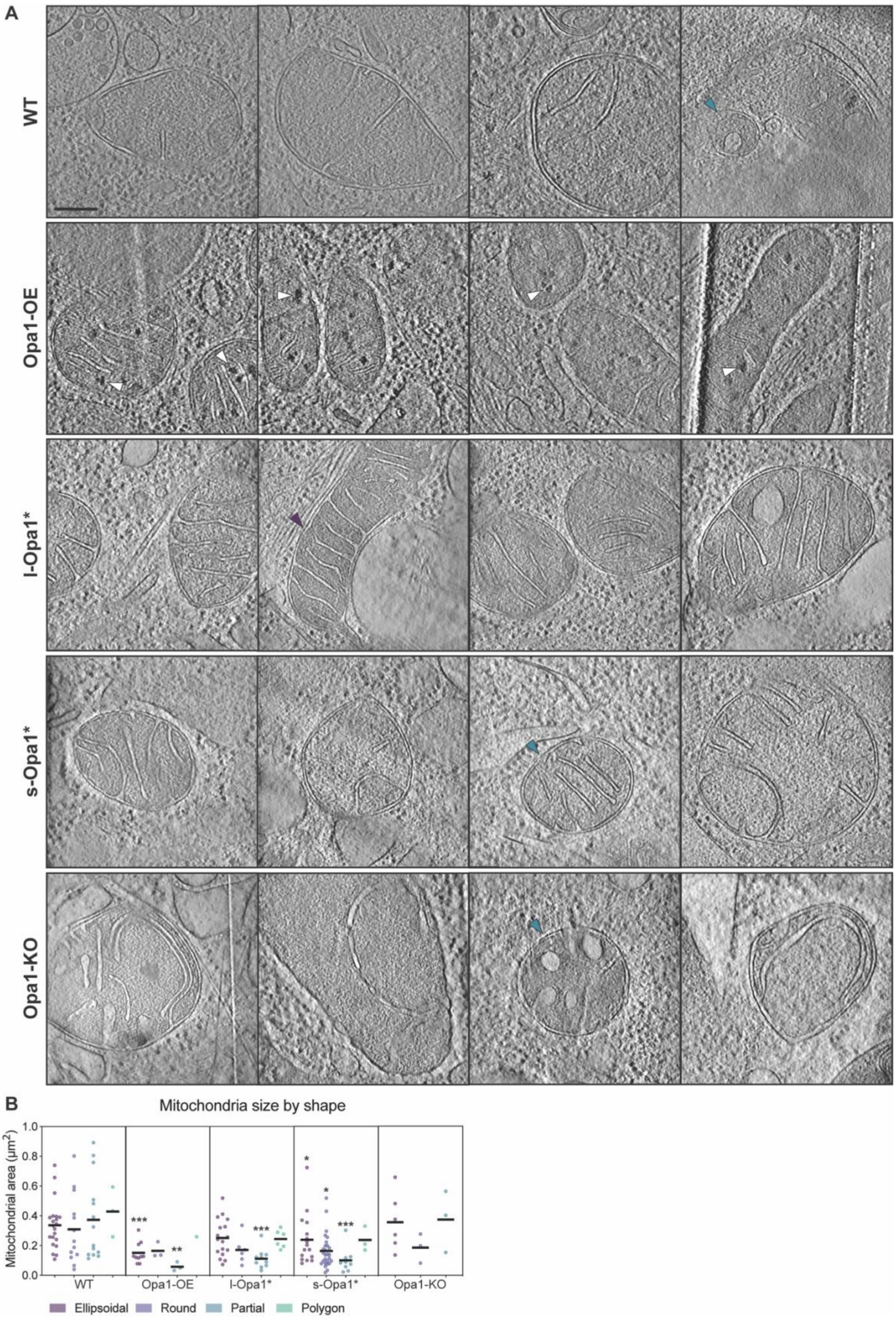
A
WT
Opa1-OE
l-Opa1*
s-Opa1*
Opa1-KO
B
Mitochondria size by shape
Mitochondrial area (µm²)
1.0
0.8
0.6
0.4
0.2
0.0
***
**
***
*
*
***
WT
Opa1-OE
l-Opa1*
s-Opa1*
Opa1-KO
Ellipsoidal
Round
Partial
Polygon

Appendix Figure S2 | Gallery of cryo-ET data.

A. Summed, projected central slices of cryo-electron tomograms visualizing mitochondria in wild-type, Opa1-OE, l-Opa1*, s-Opa1* and Opa1-KO MEF. White arrowheads indicate calcium deposits, blue arrowheads indicate ellipsoidal mitochondria and purple arrowheads indicate round mitochondria. Scale bar = 200 nm. The second projections from the left for WT and l-Opa1* the same tomograms as displayed in Fig 1a. The left most Opa1-OE projection is the same tomogram as in Fig. 1a.

B. Mitochondria size (μm^2) broken down by shape per cell line. N refers to number of mitochondria: WT = 57, Opa1-OE = 17, l-Opa1* = 39, s-Opa1* = 55, Opa1-KO = 12.

Data Information: Scatter plots show data distribution, the mean is shown by a bold black line. Significance of difference is tested relative to wild type using Mann-Whitney; *p<0.1, **p<0.01, ***p<0.001.

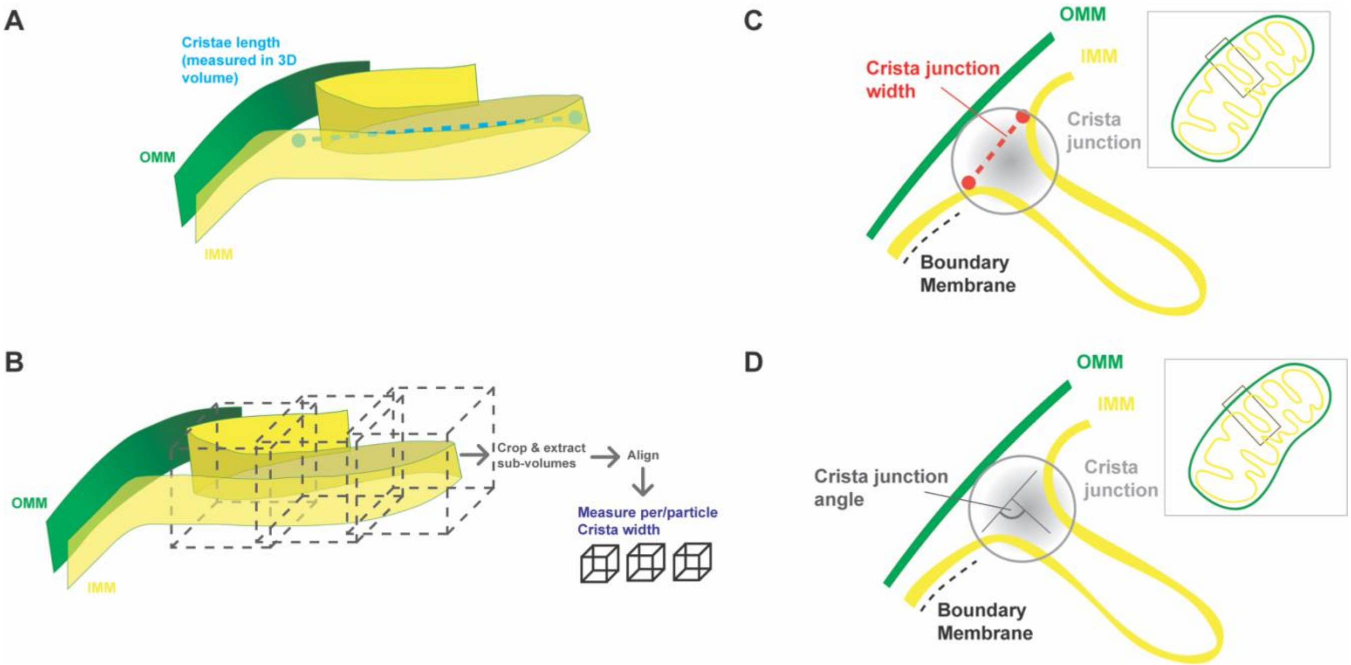

Appendix Figure S3 | Cristae length, width quantification, junction width, angle.

A. Cartoon schematic representing sub-tomogram averaging (STA) approach for measuring crista length in 3D.

B. Cartoon schematic representing sub-tomogram averaging (STA) approach for measuring crista width in 3D.

C. Cartoon schematic for measurement of cristae junction width.

D. Cartoon schematic for measurement of cristae junction width angle. See Methods for details.

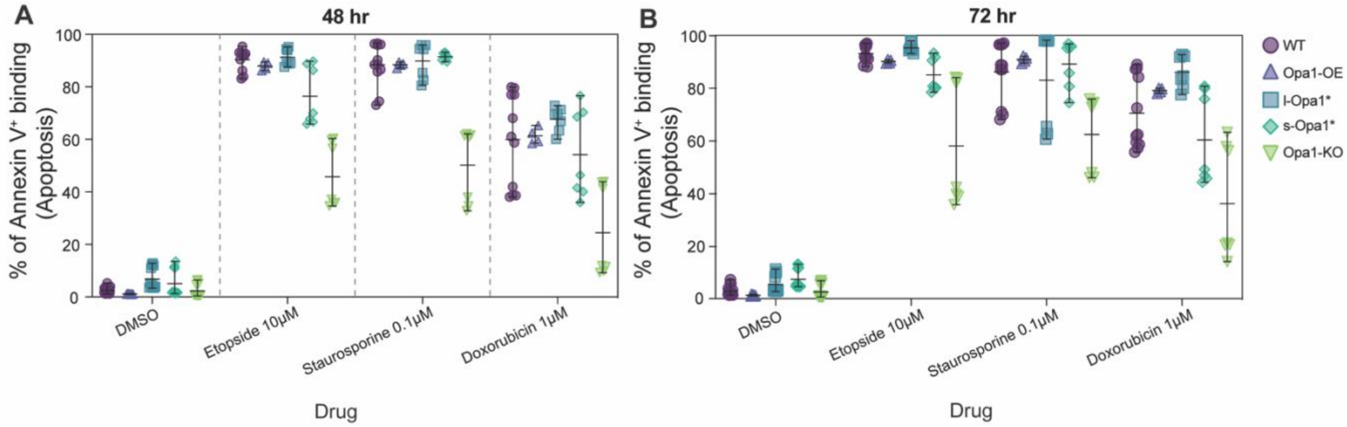

Appendix Figure S4 | Cell viability following apoptotic priming.

A. Assessment of cell viability by Annexin V staining in MEF cell lines after treatment with the indicated compounds for 48 hours. Error bars represent range of N = minimum 4 biological replicates.

B. Assessment of cell viability by Annexin V staining in MEF cell lines after treatment with the indicated compounds for 72 hours. Error bars represent range of N = minimum 4 biological replicates.

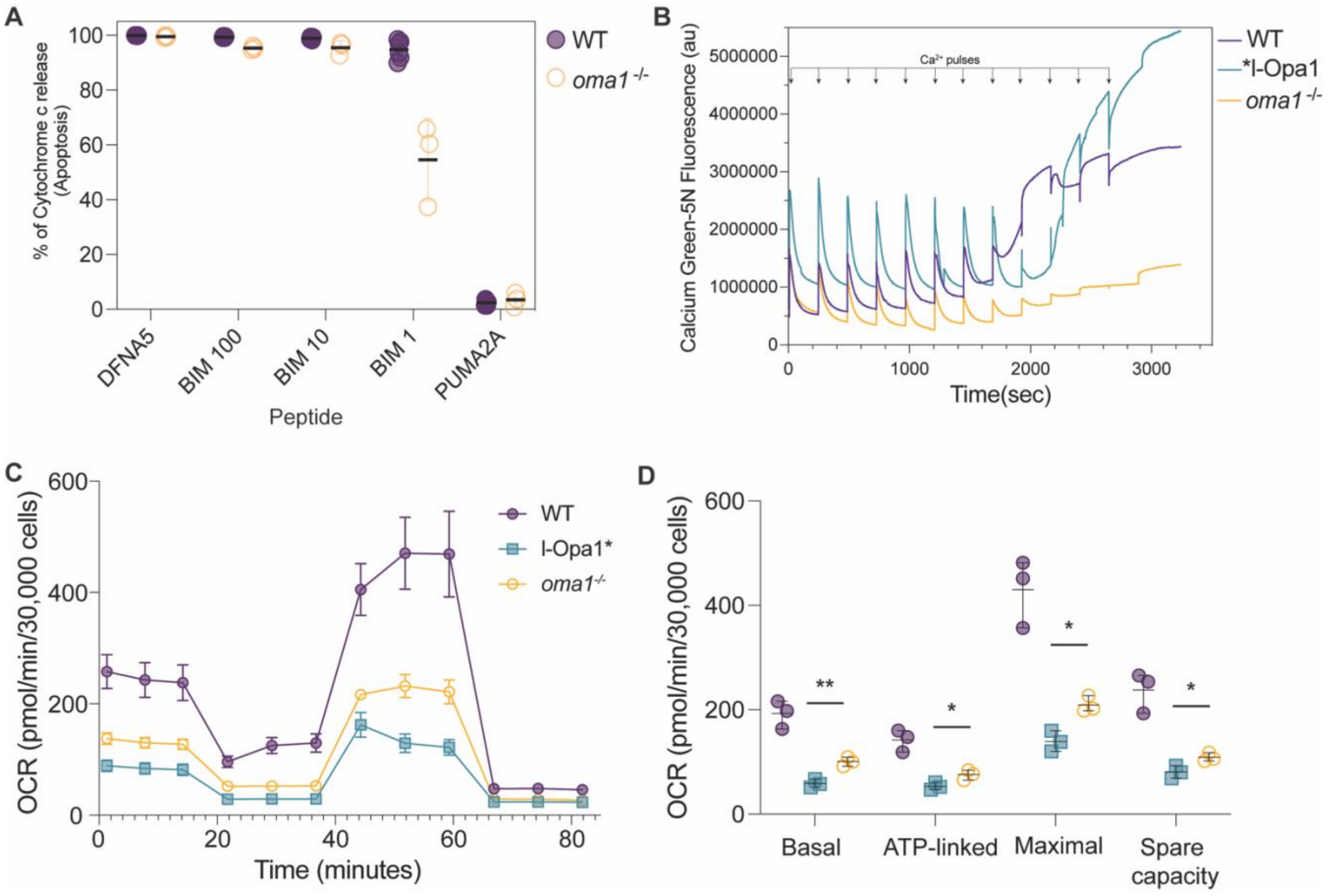

Appendix Figure S5 | *oma1*$^{-/-}$ cell functional characterization.

A. BH3 profiling of WT and *oma1*$^{-/-}$ MEF for sensitizer BIM BH3 and PUMA. N = 3 biological replicates. Error bars represent range of N = 3 biological replicates.

B. Representative traces of mitochondrial calcium retention capacity assays done in indicated MEF lines. N = 3 biological replicates.

C. OCR plotted against time for indicated MEF lines. Error bars represent SD from N = 3 biological replicates.

D. Aspects of mitochondrial respiration; basal respiration rates, the amount of respiration used for ATP production, maximum respiration, and spare capacity, are extracted by the data plotted in (c). Error bars represent range of N = 3 biological replicates.

Data Information: Significance of difference between l-Opa1* and *oma1*$^{-/-}$ is tested using Welch's t-test; *p<0.05, **p<0.01.

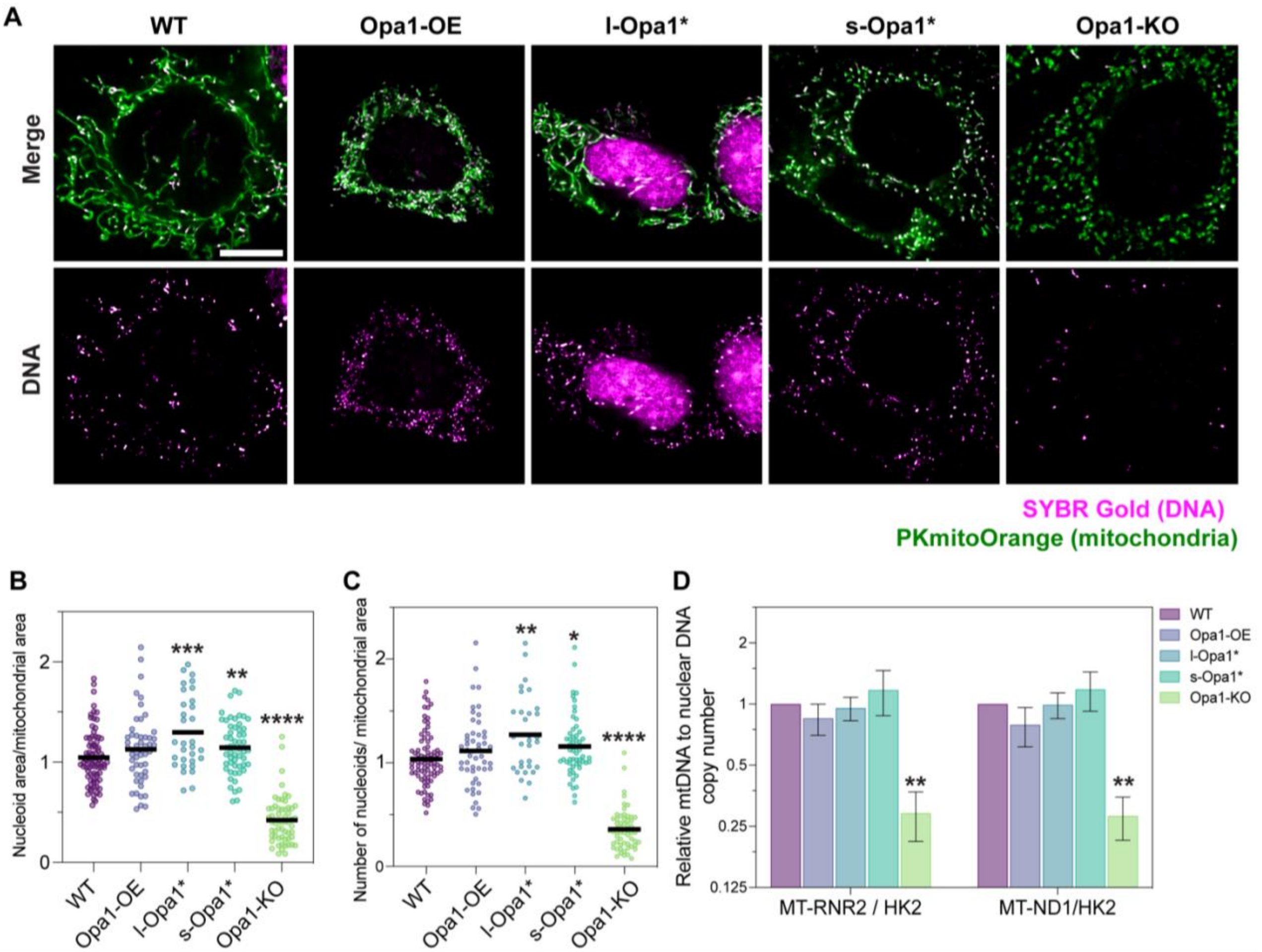

Appendix Figure S6 | mtDNA maintenance characterization.

A. Representative live iSIM images of mitochondrial network (PKmito Orange, in green) and nucleoid signal, (SYBR Gold, in magenta).

B. Quantification of mean nucleoid area normalized to mitochondrial area and relative to the experimental controls. N refers to the number of quantified cells per MEF line, WT = 83, Opa1-OE =51, l-Opa1* = 31, s-Opa1* = 55, Opa1-KO = 58.

C. Quantification of total nucleoid number per cell, normalized to mitochondrial area and relative to the experimental controls (median of WT cells imaged on the same day). N refers to the number of quantified cells per MEF line, WT = 83, Opa1-OE =51, l-Opa1* = 31, s-Opa1* = 55, Opa1-KO = 58.

D. qPCR-based determination of mtDNA (RNR2 and ND1 probes) copy number relative to nuclear genome copies (HK2 probe), normalized to WT cells. Error bars represent SD from N = 3 biological replicates.

Data Information: For (B) and (C) Significance of difference is tested relative to wild type using Mann-Whitney; *p<0.05, **p<0.01, ***p<0.001, ****p<0.0001. For (D) Significance of difference is tested relative to wild type using Welch's t-test; **p<0.001. Scale bar = 10 µm.
