# Supplementary figures and images for: In situ architecture of Opa1-dependent mitochondrial cristae remodeling
Source: EMBO J. 2024 Jan 15;43(3):391–413. doi: 10.1038/s44318-024-00027-2 (PMC10897290; doi:10.1038/s44318-024-00027-2)

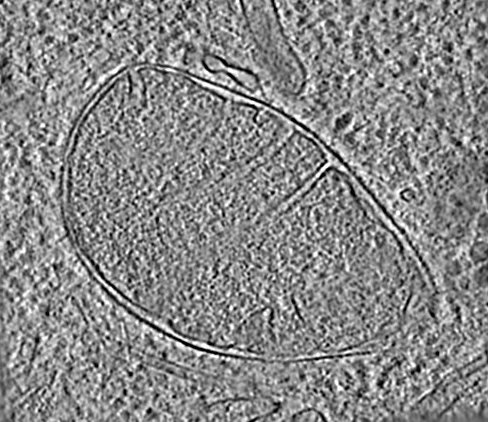

Supplement: Supplementary file 9 — Source Data Fig. 1 [file 44318_2024_27_MOESM9_ESM.zip › EMBOJ-2023-113495R_SourceDataForFig1/1A/1A_WT.tiff]

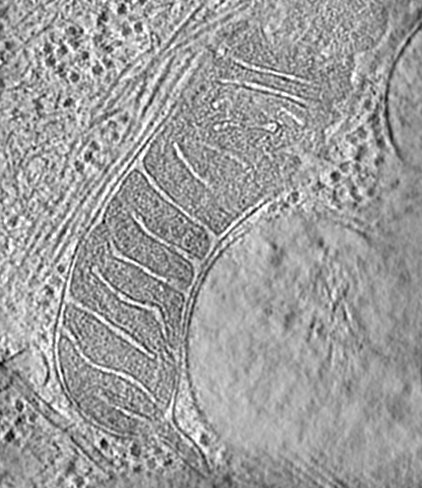

Supplement: Supplementary file 9 — Source Data Fig. 1 [file 44318_2024_27_MOESM9_ESM.zip › EMBOJ-2023-113495R_SourceDataForFig1/1A/1A_l-Opa1*.tiff]

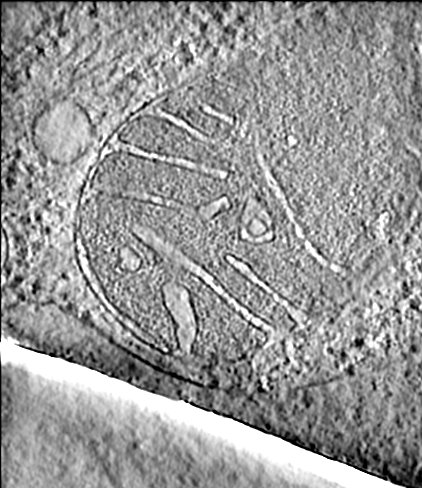

Supplement: Supplementary file 9 — Source Data Fig. 1 [file 44318_2024_27_MOESM9_ESM.zip › EMBOJ-2023-113495R_SourceDataForFig1/1A/1A_Opa1-KO.tiff]

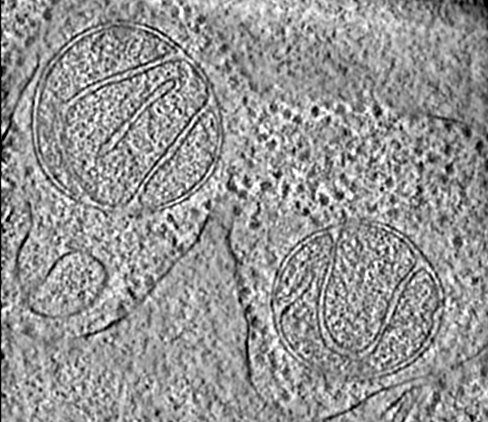

Supplement: Supplementary file 9 — Source Data Fig. 1 [file 44318_2024_27_MOESM9_ESM.zip › EMBOJ-2023-113495R_SourceDataForFig1/1A/1A_s-Opa1*.tiff]

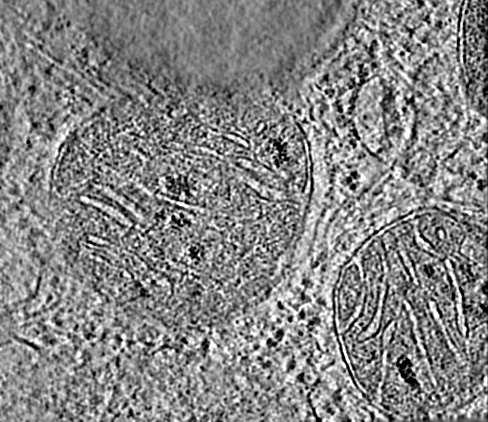

Supplement: Supplementary file 9 — Source Data Fig. 1 [file 44318_2024_27_MOESM9_ESM.zip › EMBOJ-2023-113495R_SourceDataForFig1/1A/1A_Opa1-OE.tiff]

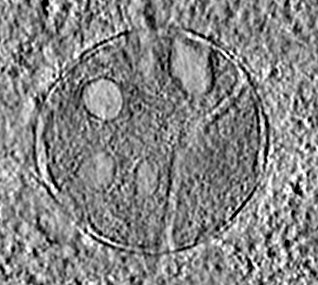

Supplement: Supplementary file 10 — Source Data Fig. 2 [file 44318_2024_27_MOESM10_ESM.zip › EMBOJ-2023-113495R_SourceDataForFig2/2E/2E_Opa1-KO_Globular.tiff]

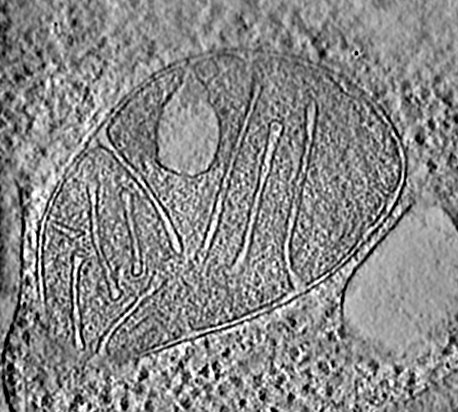

Supplement: Supplementary file 10 — Source Data Fig. 2 [file 44318_2024_27_MOESM10_ESM.zip › EMBOJ-2023-113495R_SourceDataForFig2/2E/2E_l-Opa1*_Globular.tiff]

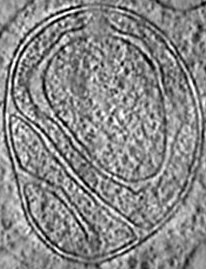

Supplement: Supplementary file 10 — Source Data Fig. 2 [file 44318_2024_27_MOESM10_ESM.zip › EMBOJ-2023-113495R_SourceDataForFig2/2E/2E_sOpa1*_Tilted.tiff]

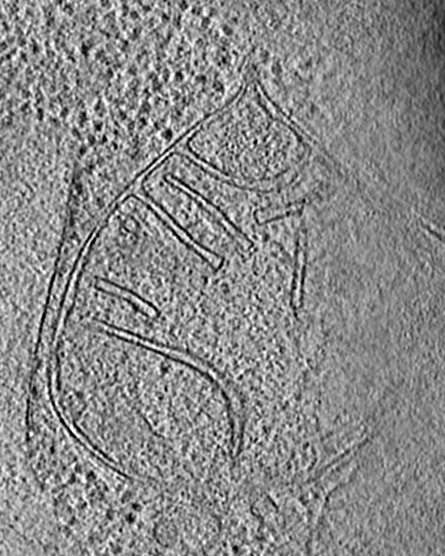

Supplement: Supplementary file 10 — Source Data Fig. 2 [file 44318_2024_27_MOESM10_ESM.zip › EMBOJ-2023-113495R_SourceDataForFig2/2E/2E_s-Opa1*_Straight.tiff]

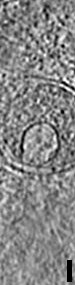

Supplement: Supplementary file 10 — Source Data Fig. 2 [file 44318_2024_27_MOESM10_ESM.zip › EMBOJ-2023-113495R_SourceDataForFig2/2E/2E_WT_globular_50nm.tiff]

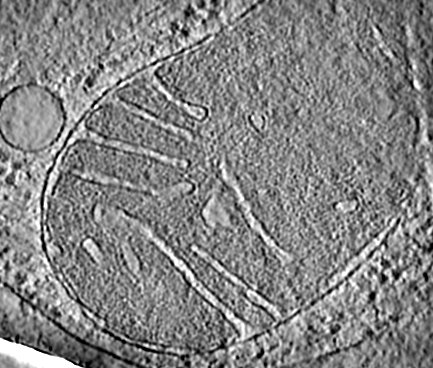

Supplement: Supplementary file 10 — Source Data Fig. 2 [file 44318_2024_27_MOESM10_ESM.zip › EMBOJ-2023-113495R_SourceDataForFig2/2E/2E_Opa1-KO_Straight.tiff]

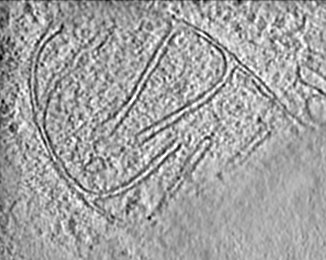

Supplement: Supplementary file 10 — Source Data Fig. 2 [file 44318_2024_27_MOESM10_ESM.zip › EMBOJ-2023-113495R_SourceDataForFig2/2E/2E_l-Opa1*_Straight.tiff]

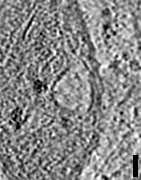

Supplement: Supplementary file 10 — Source Data Fig. 2 [file 44318_2024_27_MOESM10_ESM.zip › EMBOJ-2023-113495R_SourceDataForFig2/2E/2E_Opa1-OE_Globular_50nm.tiff]

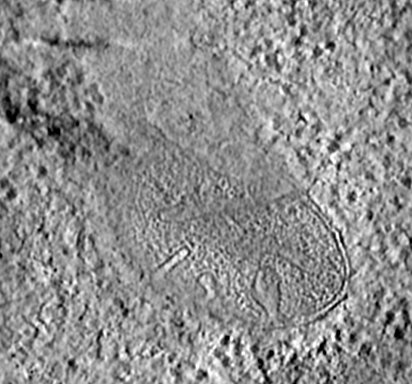

Supplement: Supplementary file 10 — Source Data Fig. 2 [file 44318_2024_27_MOESM10_ESM.zip › EMBOJ-2023-113495R_SourceDataForFig2/2E/2E_s-Opa1*_Globular.tiff]

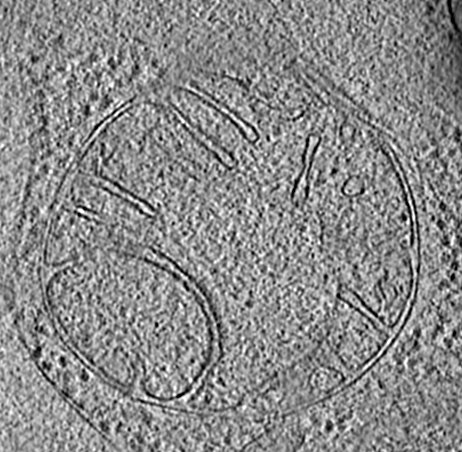

Supplement: Supplementary file 10 — Source Data Fig. 2 [file 44318_2024_27_MOESM10_ESM.zip › EMBOJ-2023-113495R_SourceDataForFig2/2E/2E_s-Opa1*_Disconnected.tiff]

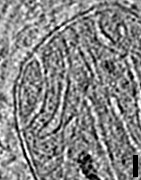

Supplement: Supplementary file 10 — Source Data Fig. 2 [file 44318_2024_27_MOESM10_ESM.zip › EMBOJ-2023-113495R_SourceDataForFig2/2E/2E_Opa1-OE_Tilted_50nm.tiff]

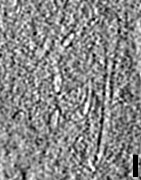

Supplement: Supplementary file 10 — Source Data Fig. 2 [file 44318_2024_27_MOESM10_ESM.zip › EMBOJ-2023-113495R_SourceDataForFig2/2E/2E_Opa1-OE_Tubular_50nm.tiff]

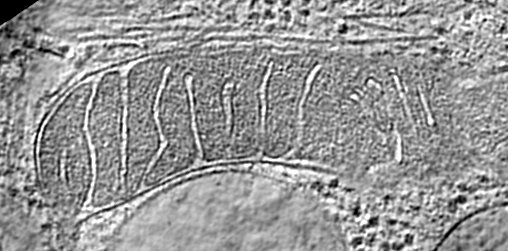

Supplement: Supplementary file 10 — Source Data Fig. 2 [file 44318_2024_27_MOESM10_ESM.zip › EMBOJ-2023-113495R_SourceDataForFig2/2E/2E_l-Opa1*_Tilted.tiff]

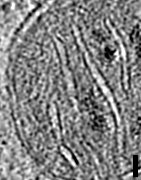

Supplement: Supplementary file 10 — Source Data Fig. 2 [file 44318_2024_27_MOESM10_ESM.zip › EMBOJ-2023-113495R_SourceDataForFig2/2E/2E_Opa1-OE_Disconnected_50nm.tiff]

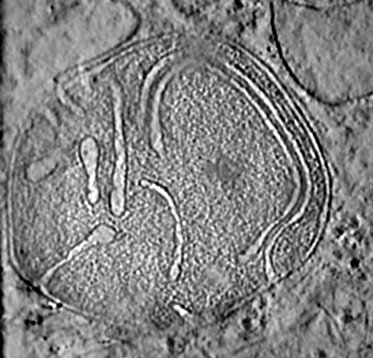

Supplement: Supplementary file 10 — Source Data Fig. 2 [file 44318_2024_27_MOESM10_ESM.zip › EMBOJ-2023-113495R_SourceDataForFig2/2E/2E_Opa1-KO_Tilted_Disconnected.tiff]

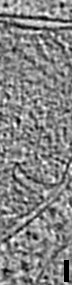

Supplement: Supplementary file 10 — Source Data Fig. 2 [file 44318_2024_27_MOESM10_ESM.zip › EMBOJ-2023-113495R_SourceDataForFig2/2E/2E_WT_Tilted_50nm.tiff]

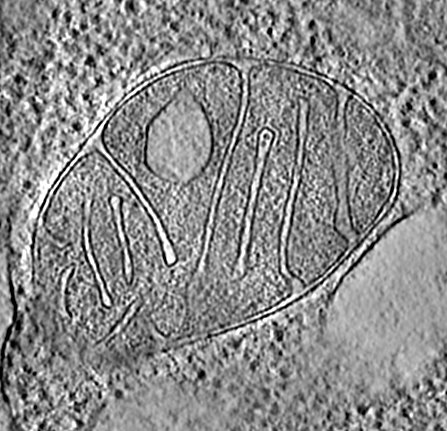

Supplement: Supplementary file 10 — Source Data Fig. 2 [file 44318_2024_27_MOESM10_ESM.zip › EMBOJ-2023-113495R_SourceDataForFig2/2E/2E_l-Opa1*_Disconnected.tiff]

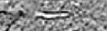

Supplement: Supplementary file 10 — Source Data Fig. 2 [file 44318_2024_27_MOESM10_ESM.zip › EMBOJ-2023-113495R_SourceDataForFig2/2E/2E_WT_Disconnected.tiff]

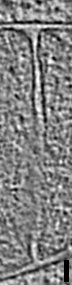

Supplement: Supplementary file 10 — Source Data Fig. 2 [file 44318_2024_27_MOESM10_ESM.zip › EMBOJ-2023-113495R_SourceDataForFig2/2E/2E_WT_straight_50nm.tiff]

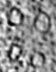

Supplement: Supplementary file 10 — Source Data Fig. 2 [file 44318_2024_27_MOESM10_ESM.zip › EMBOJ-2023-113495R_SourceDataForFig2/2E/2E_s-Opa1*_Tubular.tif]

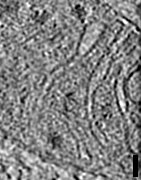

Supplement: Supplementary file 10 — Source Data Fig. 2 [file 44318_2024_27_MOESM10_ESM.zip › EMBOJ-2023-113495R_SourceDataForFig2/2E/2E_Opa1-OE_Straight_50nm.tiff]

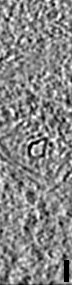

Supplement: Supplementary file 10 — Source Data Fig. 2 [file 44318_2024_27_MOESM10_ESM.zip › EMBOJ-2023-113495R_SourceDataForFig2/2E/2E_WT_Tubular_50nm.tiff]

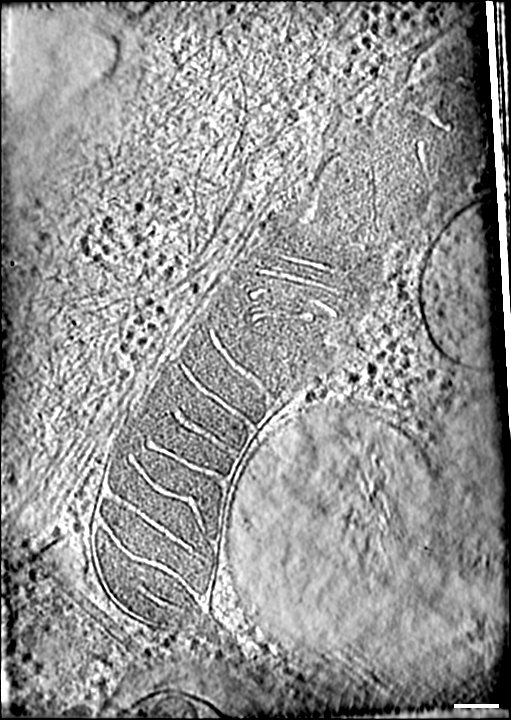

Supplement: Supplementary file 11 — Source Data Fig. 3 [file 44318_2024_27_MOESM11_ESM.zip › EMBOJ-2023-113495R_SourceDataForFig3/3C/lOpa1.tiff]

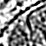

Supplement: Supplementary file 11 — Source Data Fig. 3 [file 44318_2024_27_MOESM11_ESM.zip › EMBOJ-2023-113495R_SourceDataForFig3/3C/Opa1_OE_CJ_pink.tiff]

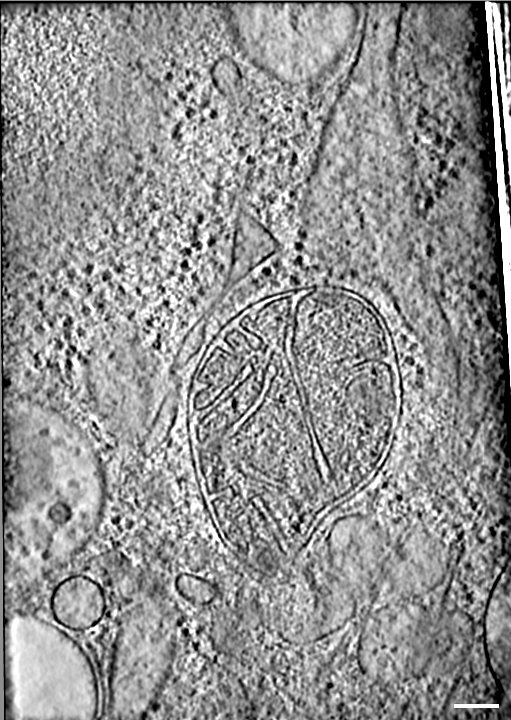

Supplement: Supplementary file 11 — Source Data Fig. 3 [file 44318_2024_27_MOESM11_ESM.zip › EMBOJ-2023-113495R_SourceDataForFig3/3C/sOpa1.tiff]

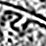

Supplement: Supplementary file 11 — Source Data Fig. 3 [file 44318_2024_27_MOESM11_ESM.zip › EMBOJ-2023-113495R_SourceDataForFig3/3C/WT_CJ_pink.tiff]

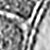

Supplement: Supplementary file 11 — Source Data Fig. 3 [file 44318_2024_27_MOESM11_ESM.zip › EMBOJ-2023-113495R_SourceDataForFig3/3C/lOpa1_CJ_cyan.tiff]

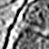

Supplement: Supplementary file 11 — Source Data Fig. 3 [file 44318_2024_27_MOESM11_ESM.zip › EMBOJ-2023-113495R_SourceDataForFig3/3C/Opa1_KO_pink.tiff]

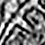

Supplement: Supplementary file 11 — Source Data Fig. 3 [file 44318_2024_27_MOESM11_ESM.zip › EMBOJ-2023-113495R_SourceDataForFig3/3C/sOpa1_CJ_cyan.tiff]

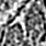

Supplement: Supplementary file 11 — Source Data Fig. 3 [file 44318_2024_27_MOESM11_ESM.zip › EMBOJ-2023-113495R_SourceDataForFig3/3C/Opa1_OE_CJ_purple.tiff]

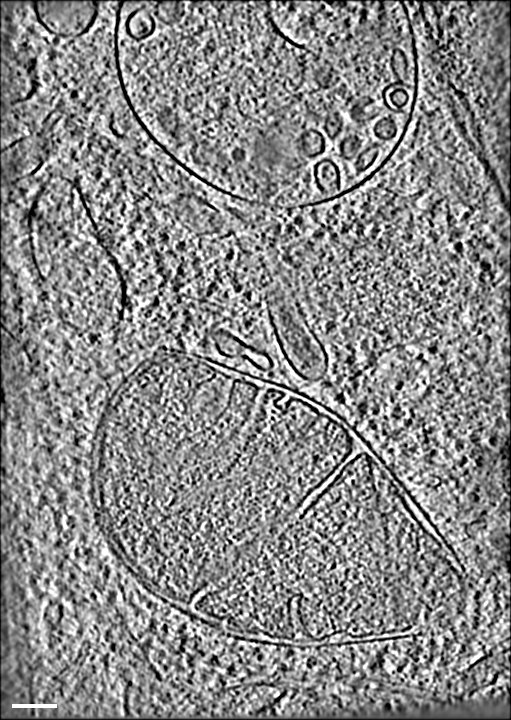

Supplement: Supplementary file 11 — Source Data Fig. 3 [file 44318_2024_27_MOESM11_ESM.zip › EMBOJ-2023-113495R_SourceDataForFig3/3C/WT.tiff]

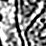

Supplement: Supplementary file 11 — Source Data Fig. 3 [file 44318_2024_27_MOESM11_ESM.zip › EMBOJ-2023-113495R_SourceDataForFig3/3C/Opa1_OE_cyan.tiff]

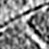

Supplement: Supplementary file 11 — Source Data Fig. 3 [file 44318_2024_27_MOESM11_ESM.zip › EMBOJ-2023-113495R_SourceDataForFig3/3C/Opa1_KO_CJ_purple.tiff]

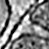

Supplement: Supplementary file 11 — Source Data Fig. 3 [file 44318_2024_27_MOESM11_ESM.zip › EMBOJ-2023-113495R_SourceDataForFig3/3C/Opa1_KO_CJ_cyan.tiff]

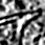

Supplement: Supplementary file 11 — Source Data Fig. 3 [file 44318_2024_27_MOESM11_ESM.zip › EMBOJ-2023-113495R_SourceDataForFig3/3C/sOpa1_CJ_pink.tiff]

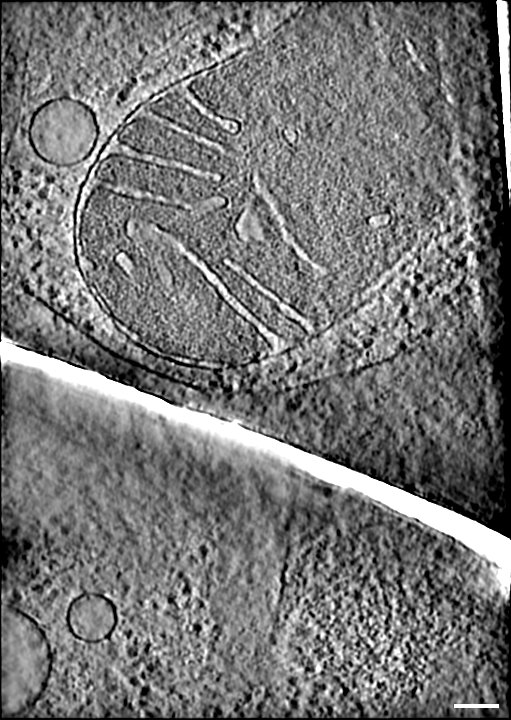

Supplement: Supplementary file 11 — Source Data Fig. 3 [file 44318_2024_27_MOESM11_ESM.zip › EMBOJ-2023-113495R_SourceDataForFig3/3C/Opa1_KO.tiff]

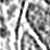

Supplement: Supplementary file 11 — Source Data Fig. 3 [file 44318_2024_27_MOESM11_ESM.zip › EMBOJ-2023-113495R_SourceDataForFig3/3C/lOpa1_CJ_pink.tiff]

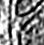

Supplement: Supplementary file 11 — Source Data Fig. 3 [file 44318_2024_27_MOESM11_ESM.zip › EMBOJ-2023-113495R_SourceDataForFig3/3C/sOpa1_CJ_purple.tiff]

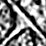

Supplement: Supplementary file 11 — Source Data Fig. 3 [file 44318_2024_27_MOESM11_ESM.zip › EMBOJ-2023-113495R_SourceDataForFig3/3C/WT_CJ_cyan.tiff]

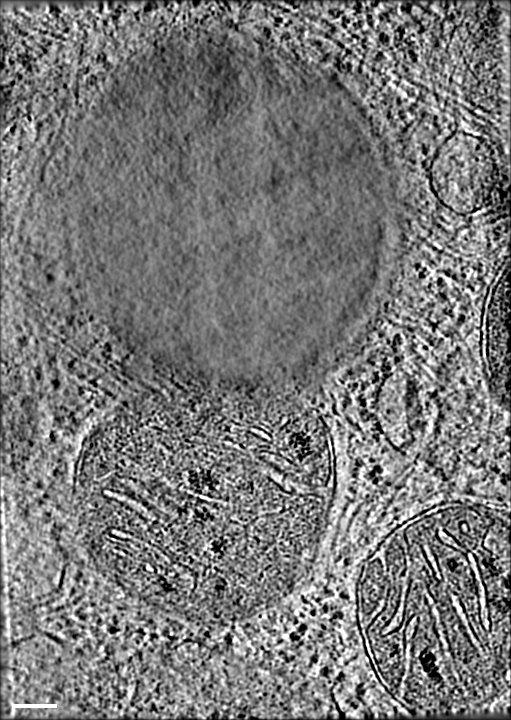

Supplement: Supplementary file 11 — Source Data Fig. 3 [file 44318_2024_27_MOESM11_ESM.zip › EMBOJ-2023-113495R_SourceDataForFig3/3C/Opa1_OE.tiff]

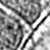

Supplement: Supplementary file 11 — Source Data Fig. 3 [file 44318_2024_27_MOESM11_ESM.zip › EMBOJ-2023-113495R_SourceDataForFig3/3C/lOpa1_CJ_purple.tiff]

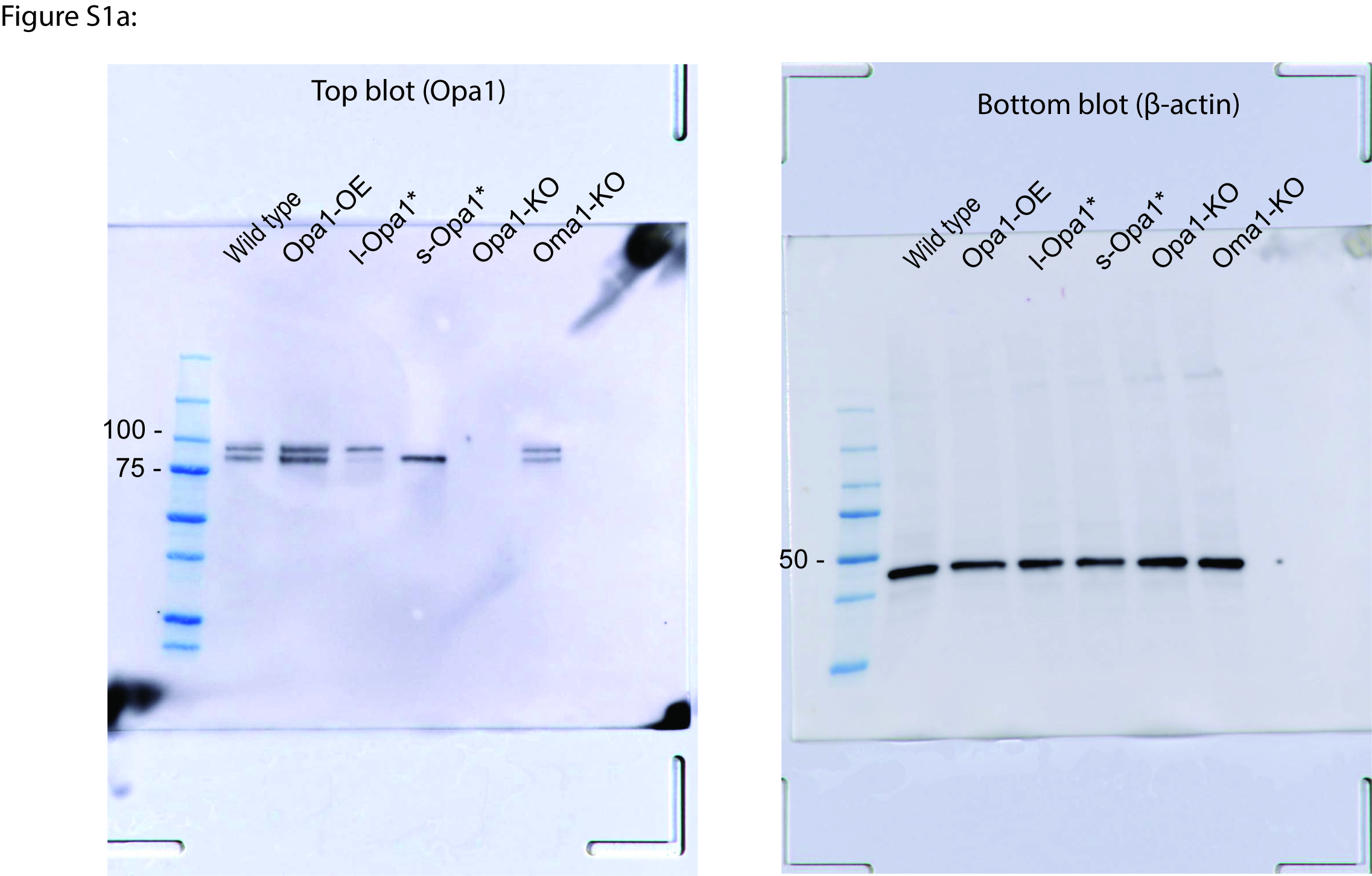

Supplement: Supplementary file 14 — Source Data Fig. S1 [file 44318_2024_27_MOESM14_ESM.zip › EMBOJ-2023-113495R_SourceDataForFigS1/FigS1_source_data.tif]
