# Supplementary material for: In situ architecture of Opa1-dependent mitochondrial cristae remodeling
Source: EMBO J. 2024 Jan 15;43(3):391–413. doi: 10.1038/s44318-024-00027-2 (PMC10897290; doi:10.1038/s44318-024-00027-2)
Supplement: Supplementary file 16 — Expanded View Figures [file 44318_2024_27_MOESM16_ESM.pdf]

Expanded View Figures

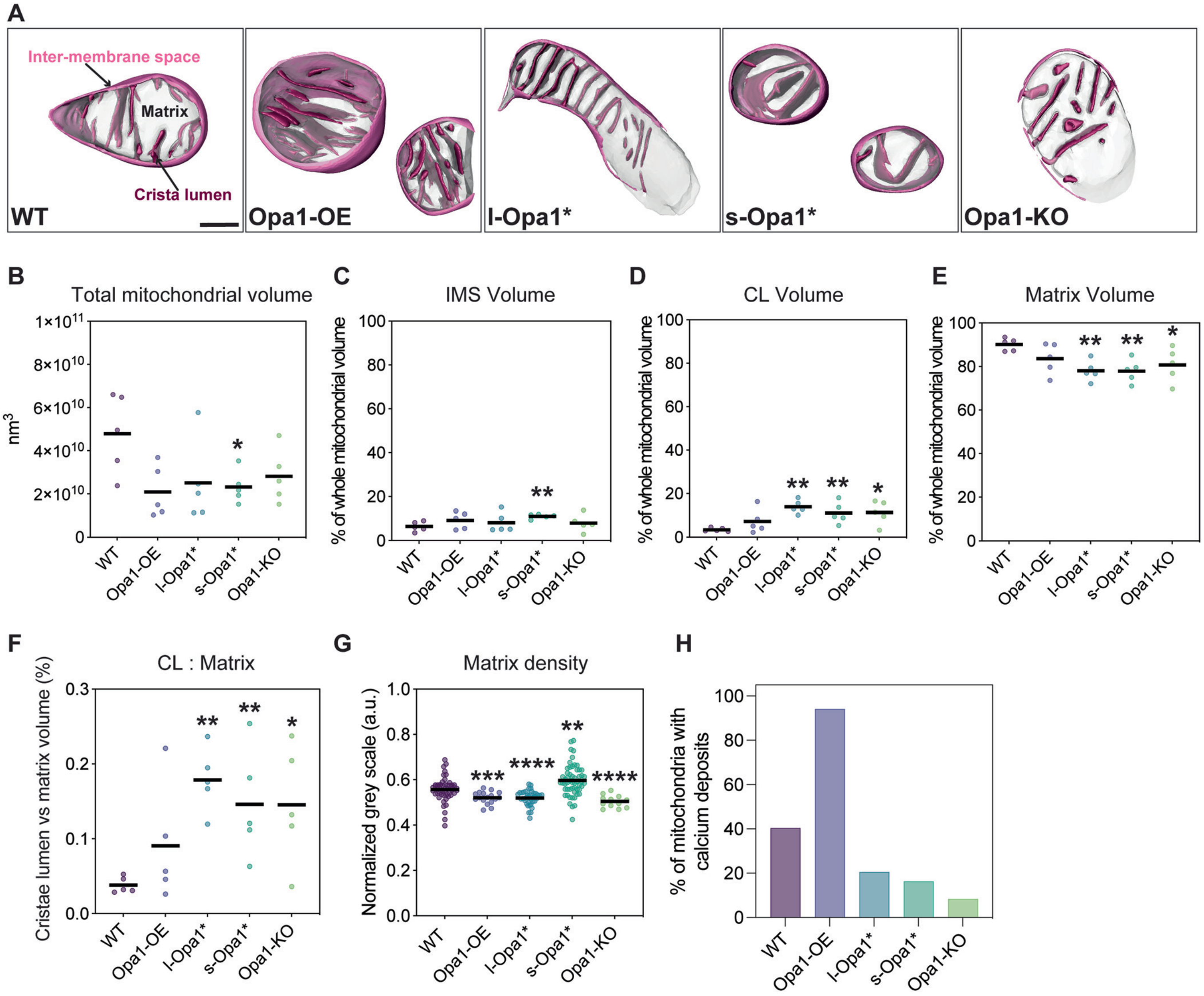

Figure EV1. Mitochondrial subcompartment volumes.

(**A**) Three-dimensional renderings of segmented inter-membrane space (IMS, pink surface), cristae lumen (CL, magenta surface), and matrix (translucent gray surface) volumes. Scale bar = 200 nm. (**B**) Total mitochondrial volume across indicated cell lines. $N = 5$ cells for all cell lines. (**C**) Quantification of IMS volume relative to total volume of each mitochondrion indicated in (**B**). $N = 5$ cells for all cell lines. (**D**) Quantification of CL volume relative to total volume of each mitochondrion indicated in (**B**). $N = 5$ cells for all cell lines. (**E**) Quantification of matrix volume relative to total volume of each mitochondrion indicated in (**B**). $N = 5$ cells for all cell lines. (**F**) CL to matrix ratio across cell lines. $N = 5$ cells for all cell lines. (**G**) Normalized gray scale mitochondrial matrix value across cell lines. $N = 5$ cells for all cell lines. (**H**) Graph bar representing percentage of cells with detected calcium deposits in cryo-electron tomograms. *N* refers to the number of mitochondria: WT = 57, Opa1-OE = 17, l-Opa1* = 39, s-Opa1* = 55, Opa1-KO = 12. Data information: Scatter plots show data distribution, the mean is shown by a bold black line. Significance of difference is tested relative to wild type using Mann–Whitney test in (**B**, **D**, **E**, **G**); $*p < 0.05$, $**p < 0.01$, $***p < 0.001$, $****p < 0.0001$; and unpaired *t* test in (**C**): $**p < 0.01$.

© The Author(s)

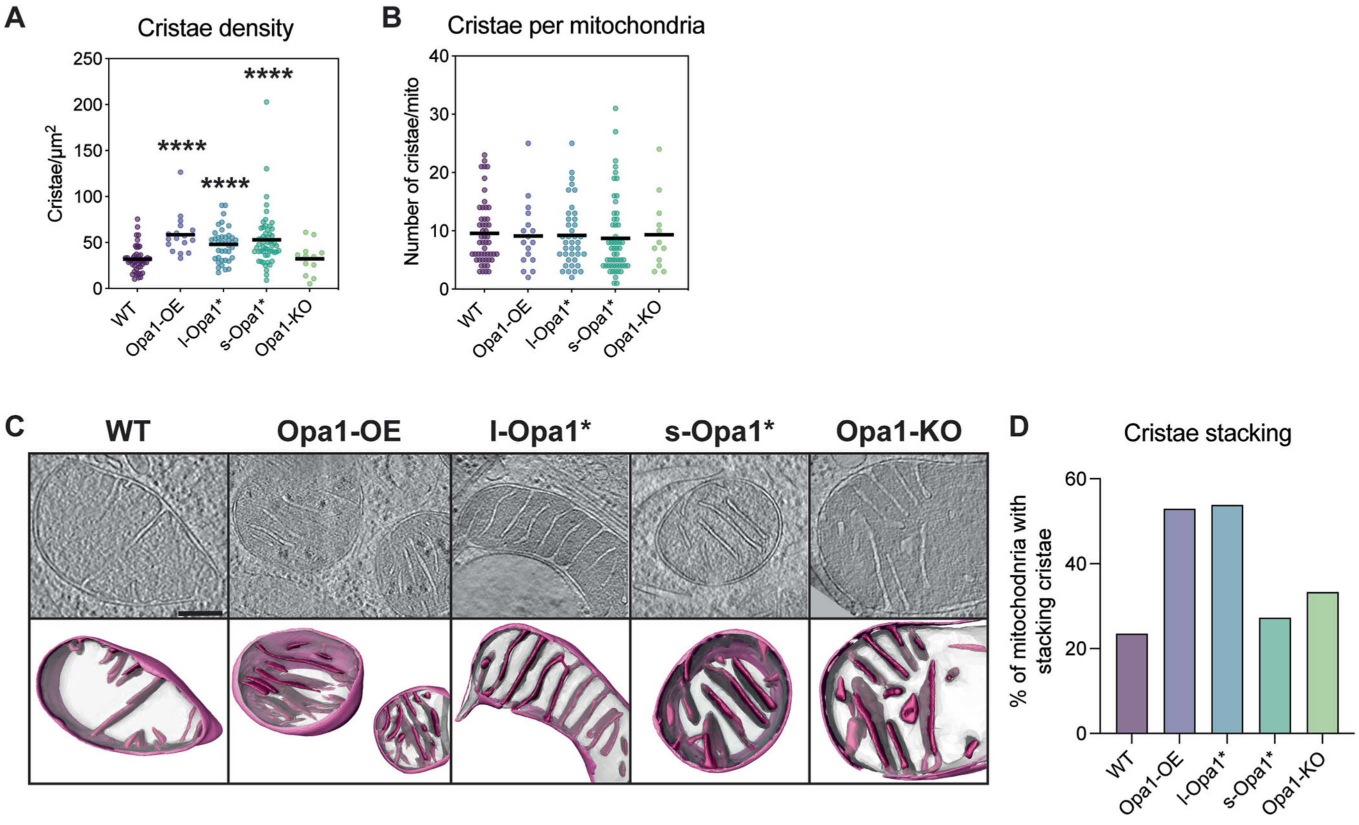

Figure EV2. Cristae analysis.

(**A**) Cristae density (cristae per μm^2) represented as a scatter plot. *N* refers to number of cells; WT = 33, Opa1-OE = 7, l-Opa1* = 21, s-Opa1* = 28, Opa1-KO = 11. (**B**) Number of cristae per mitochondria represented as a scatter plot. N refers to number of cells; WT = 51, Opa1-OE = 17, l-Opa1* = 39, s-Opa1* = 55, Opa1-KO = 12. (**C**) (Top) Summed, projected central slices of cryo-electron tomograms visualizing mitochondria with stacking crista characteristics, supported by 3D representations consisting of their sub compartments (bottom) in indicated MEF lines. Scale bar = 200 nm. *N* refers to number of cells; WT = 57, Opa1-OE = 17, l-Opa1* = 39, s-Opa1* = 55, Opa1-KO = 12. The representative tomograms for WT, Opa1-OE, l-Opa1*, and Opa1-KO are the same as in Fig. 1A. The representative s-Opa1* tomogram is the same as the second from the left in Appendix Fig. S2A. (**D**) Graph bar representing percentage of mitochondria with stacking crista formation in each MEF line. *N* refers to number of cells; WT = 57, Opa1-OE = 17, l-Opa1* = 39, s-Opa1* = 55, Opa1-KO = 12. Data information: Significance of difference is tested relative to WT using Mann–Whitney test; ****$p < 0.0001$.

© The Author(s)

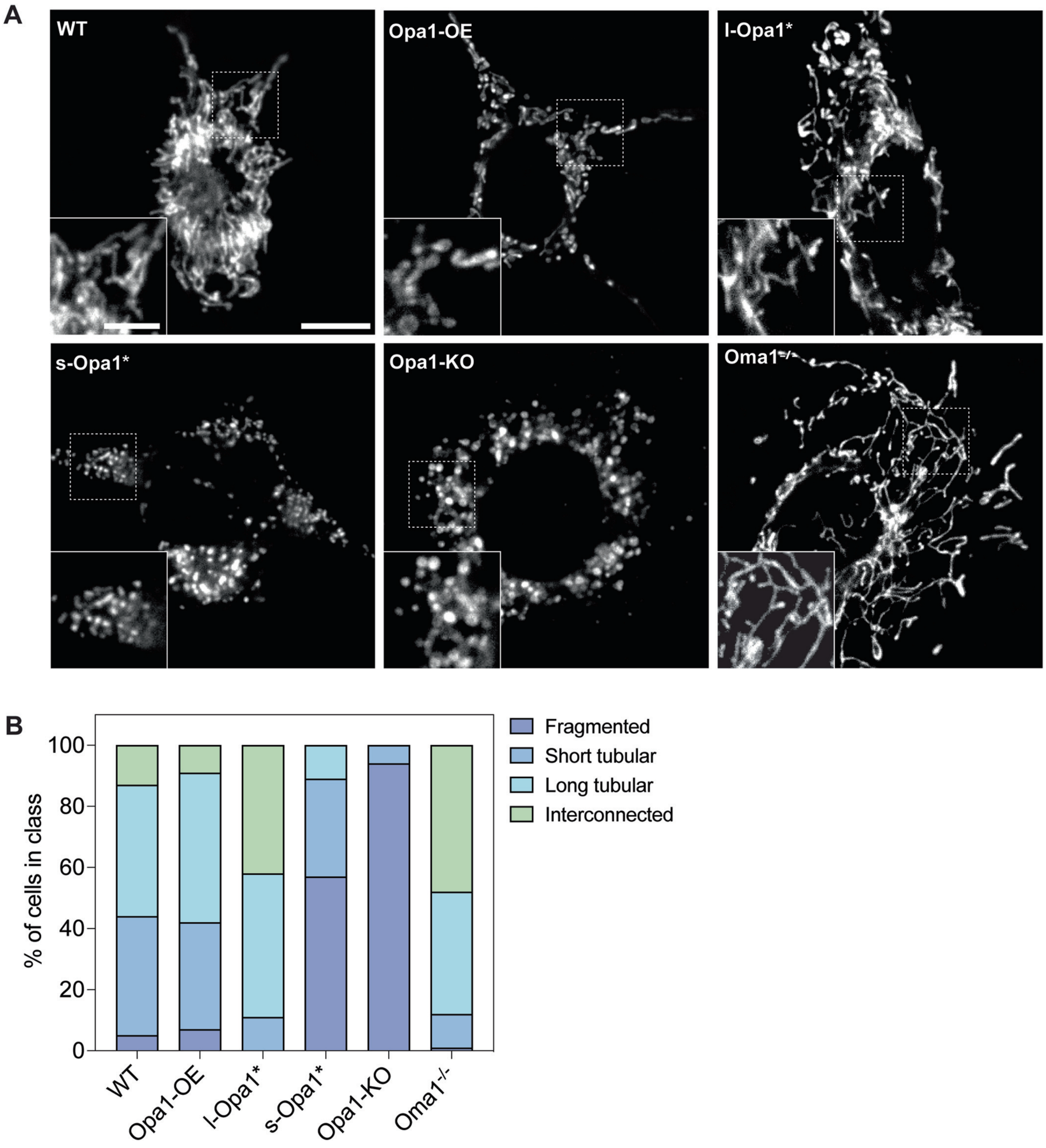

Figure EV3. Mitochondrial network morphology in MEF lines by fluorescence microscopy.

(**A**) Representative images of mitochondrial morphology in indicated MEF lines labeled with MitoTracker^{TM} Deep Red FM. Insets show magnified view of regions indicated with dashed boxes. Scale bar = 10 µm. Inset scale bar = 5 µm. (**B**) Graph bar representing mitochondrial network morphology scored in indicated MEF lines. $N = 100$ cells analyzed per cell line.

 © The Author(s)

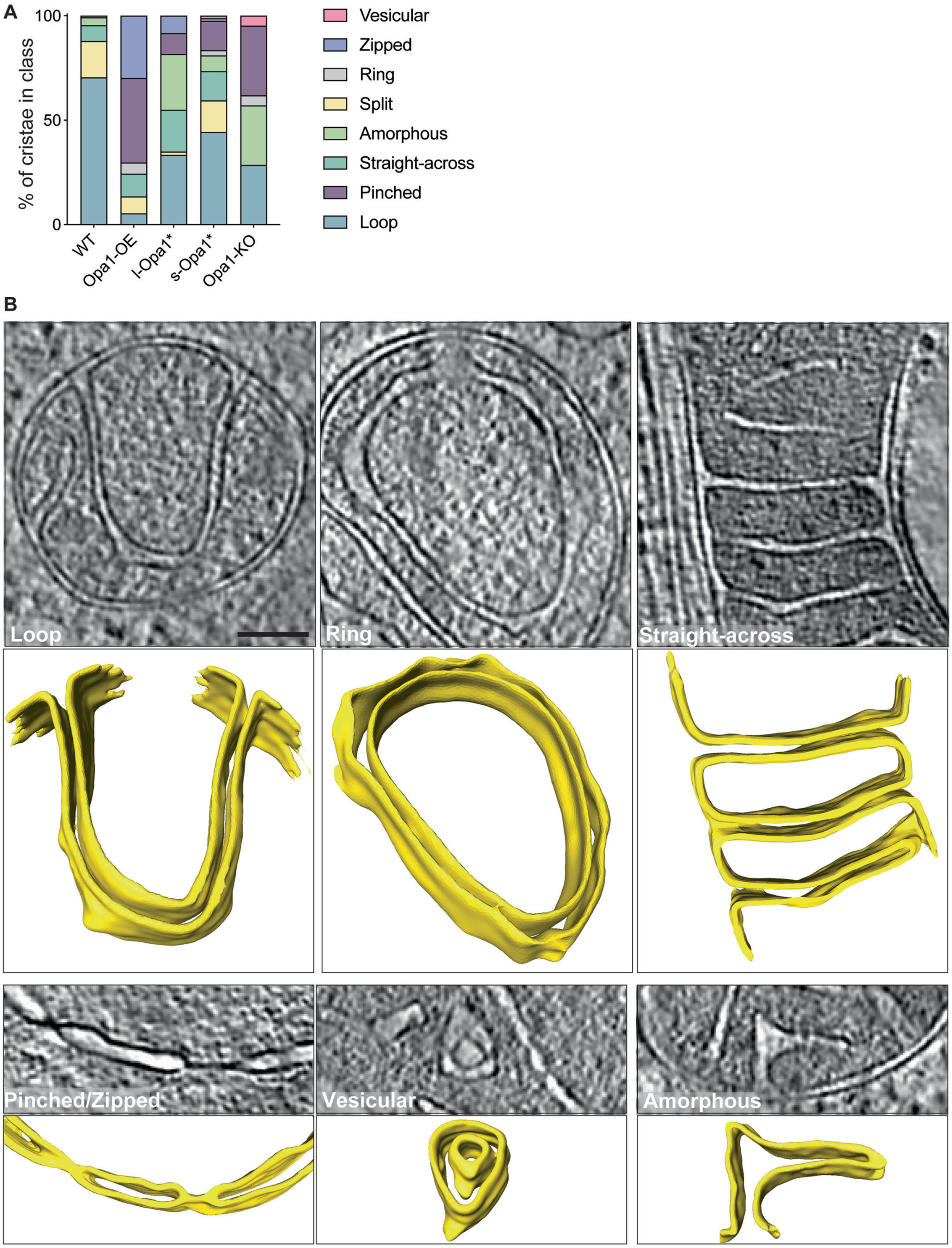
A
100
50
0
% of cristae in class
WT
Opa1-OE
l-Opa1*
s-Opa1*
Opa1-KO
Vesicular
Zipped
Ring
Split
Amorphous
Straight-across
Pinched
Loop
B
Loop
Ring
Straight-across
Pinched/Zipped
Vesicular
Amorphous

Figure EV4. Unusual cristae morphology.

(**A**) Graph bar representing the relative proportion of unusual cristae morphology observed in indicated MEF lines. Unusual cristae were categorized into vesicular, zipped, ring, split, amorphous, straight-across, pinched and loop. *N* refers to number of cristae analyzed, *N*: wild-type = 222, Opa1-OE = 430, l-Opa1* = 323, s-Opa1* = 653, Opa1-KO = 243. (**B**) Summed, projected central slices of cryo-electron tomograms showing examples of unusual cristae in mitochondria across cell lines in 2D (top) and 3D (bottom). Loop (from Fig. 1A, s-Opa1*), ring, straight-across (from Fig. 1A, l-Opa1*), pinched (from Appendix Fig. S2A, Opa1-KO second from the left), vesicular (from Fig. 1A, Opa1-KO), and amorphous cristae are shown. Scale bar = 200 nm.

© The Author(s)

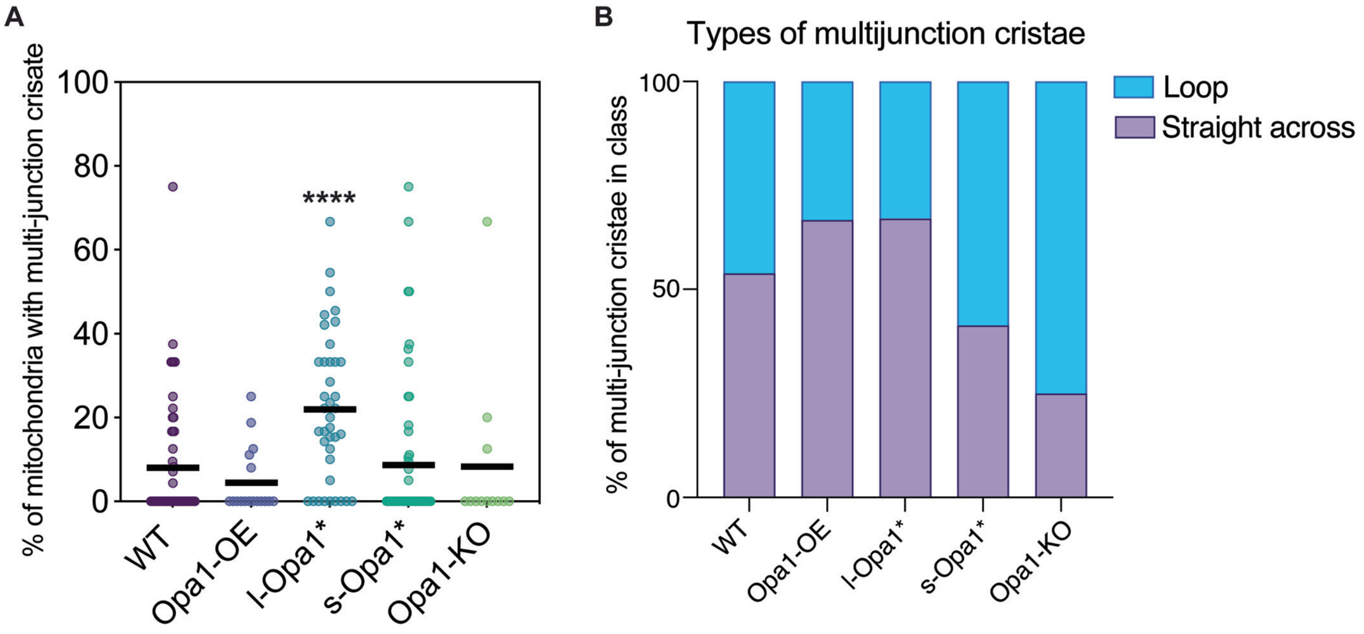

Figure EV5. Multijunction cristae.

(**A**) Scatter plot showing the percentage of multijunction cristae per mitochondrion in indicated MEF lines. *N* refers to number of cristae; WT = 18, Opa1-OE = 5, l-Opa1* = 30, s-Opa1* = 16, Opa1-KO = 3. (**B**) Graph bar representing percentage of multijunction cristae categorized into straight-across and loop morphology in each MEF line. *N* refers to number of cristae; WT = 26, Opa1-OE = 9, l-Opa1* = 79, s-Opa1* = 29, Opa1-KO = 4. Data information: Scatter plot shows data distribution, the mean is marked by a bold black line. Significance of difference is tested relative to wild type using Mann–Whitney; ****$p < 0.0001$.
